# The Dark Triad of personality and attitudes toward cognitive enhancement

**DOI:** 10.1186/s40359-020-00486-2

**Published:** 2020-11-07

**Authors:** Eric Mayor, Maxime Daehne, Renzo Bianchi

**Affiliations:** 1 Department of Clinical Psychology and Epidemiology, University of Basel, 4055 Basel, Switzerland; 2 Institute of Work and Organizational Psychology, University of Neuchâtel, 2000 Neuchâtel, Switzerland

**Keywords:** Cognitive enhancement, Public attitudes, Dark triad of personality, Competitiveness, Ethical views

## Abstract

**Background:**

Cognitive enhancement (CE) refers to the voluntary improvement of human cognitive capabilities. Few studies have examined the general attitude of the public towards CE. Such studies have suggested that the use of CE is considered largely unacceptable by the public. In parallel, past research indicates that individuals scoring high on the Dark Triad of personality (Machiavellianism, narcissism, and psychopathy) and competitiveness have atypical views of ethical questions. In this study, we examined (a) whether attitudes towards CE are associated with individual differences in the Dark Triad of personality as well as in trait and contextual competitiveness and (b) whether the Dark Triad moderates the effect of trait and contextual competitiveness on attitudes towards CE.

**Method:**

US employees (*N* = 326) were recruited using Mechanical Turk. Participants completed a web survey. Data were analyzed by means of (robust) hierarchical regression and (robust) ANCOVAs.

**Results:**

The Dark Triad of personality and one of its subscales, Machiavellianism, predicted positive attitudes towards CE. Neither trait competitiveness nor contextual competitiveness were linked to general attitudes towards CE, but the DT was a positive moderator of the association between contextual competitiveness and positive attitudes.

**Conclusion:**

Our findings extend the incipient knowledge about the factors relating to favourable views of CE by highlighting the role of dark personality traits in shaping such views. Our study further shows contextual factors can play a differentiated role with respect to such attitudes depending upon dark personality traits. Implications for policy-making are discussed.

## Background

Cognitive enhancement (CE) refers to the voluntary improvement of human cognitive capabilities [1, 2]. Due to increased exposure of the public to this topic in recent years and concerns regarding an expanding adoption of CE, governments of several countries mandated expert reports and drafted policies regarding CE [3]. During the last decade, the public has been largely exposed to the topic of CE due to a persistent interest in CE in the entertainment industry (e.g., movies such as “Limitless”, the Netflix show “Take Your Pills”) and in the press [4]. The media generally depicted CE as widespread, efficient, and safe, which is inconsistent with research findings (e.g., [5, 6]). But public understanding is itself composed of a diversity of views and most often, studies interested in the content of attitudes towards cognitive enhancement (for a review, see [7]) have paid little attention to the personal and contextual factors associated with attitudes towards CE.

Following recent calls for a better understanding of public views of human enhancement (e.g., [8]), we examined the role of the Dark Triad of personality (DT), trait competitiveness, and intra-organizational competitive climate in shaping positive attitudes towards the use of pharmaceutical cognitive enhancers, otherwise known as smart drugs. The DT and competitiveness have been associated with atypical ethical positions [9–11], a characteristic that may be reflected in participants’ attitudes towards CE.

## Cognitive enhancement

CE can be divided into two broad subcategories: Non Pharmacological Cognitive Enhancement (NPCE) and Pharmacological Cognitive Enhancement (PCE). NPCE includes a variety of methods that do not entail recourse to legal or illegal drugs. These methods notably include tea and coffee consumption, having a healthy diet and consuming dietary supplements (for a review, see [12]). PCE refers to the use of legal or illegal drugs for the purpose of improving cognitive performance (for a review, see [2]).

The prevalence of use of PCE varies depending on the context (ranging from 1 to 55%), and is especially high among some professional groups and students [13–15]. While research shows that medical risks associated with PCE outweigh its benefits [5, 6, 16–18], the public overestimates the effectiveness of PCE and underestimates the risks of undesirable side effects [15, 19, 20]. A study analyzing data from 15 countries participating in the General Drug Survey, show that the use of PCE has increased multiple fold from the 2015 edition to that of 2017 in several countries and decreased in none [21].

Based upon regulatory, ethical and public health considerations several reasons have been advanced for forbidding PCE use (e.g., [22–24]). But there are disagreements as to whether such prohibition would effectively change practices (e.g., [22, 24]).

Only few studies have investigated the attitudes of the general public towards CE (see [7]). While NPCE is primarily considered a legitimate means to protect one’s cognitive resources [25], PCE is thought to reduce the authenticity of the performance of its users [26, 27]. Several quantitative studies found that the general public is against the use of PCE, particularly when PCE is perceived to entail health risks and when adolescents and children are the users [28–30]. Yet, higher familiarity with PCE is linked to more favourable normative attitudes towards PCE [29]. Conrad and colleagues [31] have shown that the framing of cognitive enhancement as fuel versus steroid as well as context of use impacts the attitudes towards cognitive enhancement.

The attitudes of the student population were investigated more extensively (for a review, see [7]). Researchers have observed the normalisation of legal and prescribed substances to help students study (but not illegal substances) and that, on average, students do not judge the use of such drugs as either moral or immoral [32, 33]. Yet students’ opinions on this topic are ambivalent [34, 35]. Erasmus and Kotze [36] noted that a majority of medical students viewed the use of PCE as potentially effective but also harmful and unfair. Contextual and substance characteristics are related to students’ ethical judgement towards PCE [37], and students judged the use of PCE as less ethical when it entails long-term health consequences [33]. Subsequently, students are more willing to use cognitive enhancers that are associated with fewer health risks and are more effective [38].

## The current study

Quantitative research on the factors related to positive attitudes towards PCE has been sparse [29, 33, 37]. However, identifying such factors is of paramount importance because positive attitudes influence actual behaviour [39] that can be harmful as in the case of PCE (see [17]). A recent study has shown that 21% of variance in the assessment of attitudes towards individual cognitive enhancers lies at the level of participants [40].

Individuals holding positive attitudes towards PCE are in the minority. For instance, in a Swedish study, 84% of respondents found it unacceptable to use PCE [28]. In a German student sample, 75% of non-users disapproved the use of PCE by students with low academic performance [41]. Overall, the public tends to consider the use of PCE to involve a loss of performance authenticity, to be unfair to those who do not use PCEs, and to be a form of cheating [26, 42].

In this study, we examined determinants of positive attitudes towards PCE. More specifically, we investigated whether positive attitudes towards PCE are related to the composite index of the DT and its dimensions, consisting of Machiavellianism, narcissism, and psychopathy [43]. We also examined the relationship of trait and contextual competitiveness with such attitudes as DT and competitiveness are related to atypical ethical positions. Finally, we tested the moderating effect of the DT on such relationships.

### The Dark Triad of personality

DT traits might have evolved as “a coordinated system of specialized adaptations for exploiting socioecologies” [44, p. 28, 45, 46]. Individuals endorsing DT traits have a preference for strong hierarchies and exhibit a propensity to seek high social status and personal gain at the expense of others [47, 48]. They hold unusual attitudes and tend to be morally disengaged; they approve ideas that other people would generally reject as morally unacceptable, and easily find exceptions to the applicability of commonly accepted moral principles [9]. This could be explained by a lack of self-conscious emotions (notably shame and guilt) in the DT of personality; [49, 50]). Indeed, Machavellians and psychopaths show deficits in these emotions [50] (but see [51]) and most empirical research also finds non-pathological narcissism to be negatively related to shame (and positively with shame avoidance; e.g., [52–56].

Individuals endorsing DT traits act deceptively, nefariously and criminally more frequently than others [55, 57, 58]. Psychopathy is notably associated with drug use [59], Machiavellianism with unethical choices [60], and narcissism with risk-taking [49] and unethical decisions and behaviour as well [55]. Machiavellianism and psychopathy are associated with cutting corners at work [61]. All three DT dimensions are related to workplace manipulation tactics and positive attitudes towards doping in sports [62, 63]. Finally, Machiavellianism is associated with the use of PCE [64] and narcissists have a propensity for self-enhancement [65, 66]. In sum, DT is a likely explanatory factor in positive attitudes towards PCE because on the one hand holding such attitudes is atypical and a large majority of individuals find that there are ethical issues regarding the use of PCE, and on the other hand a strong endorsement of DT traits is related to self-serving behaviour, seeking social status—which the public thinks PCE can help attaining, and an impaired ability to feel negative self-conscious emotions. On this basis, we made the following hypotheses.

#### Hypothesis 1:

The DT composite index is positively related to favourable general attitudes towards PCE.

#### Hypothesis 2:

Machiavellianism is related to favourable general attitudes towards PCE.

#### Hypothesis 3:

Psychopathy is related to favourable general attitudes towards PCE.

#### Hypothesis 4:

Narcissism is related to favourable general attitudes towards PCE.

### Trait competitiveness and competitive climate

Competition can be viewed as a quest for status stemming from a need for social acceptance [67]. Following this line of reasoning, higher attempts at increasing status could be observed in competitive contexts and from competitive individuals. There are dark sides to competition, notably an increase in negative self-conscious emotions such as shame in case of failure or when failing is a risk [68]. The research literature has recently distinguished between internal shame (self-evaluation) and external shame (perception of others as critical to oneself; [67]). Such distinction has been shown to be relevant with regards to physiological reactions as well as mental health [69, 70]. Shame (external shame particularly, [71]; but also internal shame, [72]), result from the perception of failure in attempts to increase or maintain positive views of the self or in attempts to distance from negative views of the self [68, 73, 74].

Highly competitive individuals and people involved in high-competition contexts are more likely to set performance-oriented goals [75] and to have atypical views on ethics, such as declaring acceptable to ‘do anything to win’ [11]. Thus, individuals scoring high on trait competitiveness might consider that using PCE is a suitable way of reaching performance-oriented goals and assess PCE positively.

Smith and Hogg [76] insisted on the importance of the social context in the formation of attitudes and the performance of behaviour. Highly competitive climates have been associated with a variety of dishonest conducts in employees and students. For instance, a more competitive climate is related to more faking during job interviews [77] and more cheating from students [78]. Our hypotheses also draw on the reported higher use of PCE in competitive contexts [79]. Building up on past research, we made the following hypotheses:

#### Hypothesis 5:

Trait competition is positively related to positive attitudes towards PCE.

#### Hypothesis 6:

Competitive climate is positively related to positive attitudes towards PCE.

### The dark triad as a moderator of the effect of trait competitiveness and competitive climate

The effect of trait competitiveness and competitive climate on positive attitudes towards PCE could be made more salient in individuals endorsing DT traits, as they have an increased motivation to gain status and avoid negative self-conscious emotions [47, 52, 66, 80]. We therefore propose:

#### Hypothesis 7:

The DT composite index positively moderates the positive relationship of trait competitiveness and competitive climate with positive attitudes towards PCE: these relationships are stronger for individuals high in DT compared to individuals low in DT.

#### Hypothesis 8:

Machiavellianism positively moderates the positive relationship of trait competitiveness and competitive climate with positive attitudes towards PCE: these relationships are stronger for individuals high in Machiavellianism compared to individuals low in Machiavellianism.

#### Hypothesis 9:

Psychopathy positively moderates the positive relationship of trait competitiveness and competitive climate with positive attitudes towards PCE: these relationships are stronger for individuals high in Psychopathy compared to individuals low in Psychopathy.

#### Hypothesis 10:

Narcissism positively moderates the positive relationship of trait competitiveness and competitive climate with positive attitudes towards PCE: these relationships are stronger for individuals high in Narcissism compared to individuals low in Narcissism.

## Method

### Participants and procedure

Professionally active US residents were eligible to participate in this study. A sample of 326 employees (84.7% employed full time, 15.3% part-time; 50.3% women) were recruited using Amazon Mechanical Turk (see [81]). Data from *Turkers* has similar characteristics to datasets collected through traditional methods [81].

This study was carried out in accordance with the Declaration of Helsinki and its amendments as well as the ethical guidelines of the Swiss Psychological Society. Because this study did not involve an experimental manipulation and because there are no foreseeable risks involved, we did not seek approval from an ethics committee.

Participants completed a short online questionnaire in exchange for 0.75 USD (equivalent to 8 USD / hour). Participants were on average 38.36 years old (*SD* = 10.49). The first page of the questionnaire contained an informed consent form explaining that this study was aimed at understanding what people think of cognitive enhancers and that participants would provide information on this topic, reply to questions about themselves and their work context. It was further explained that participation to the study was anonymous. All participants provided their informed consent.

### Measures

Items were measured using a Likert scale ranging from 1 = Strongly agree, to 5 = Strongly disagree for all scales except for the competitive climate scale, which was measured on a range from 1 = Strongly disagree to 7 = Strongly agree. For all measures, relevant items were reverse-coded and was good (see Table 1). Each scale was composed by aggregation of its items (see “Appendix A” for the items).

**Table 1:** Descriptive and zero-order correlations between study variables (*N* = 326)

		*M*	*SD*	1	2	3	4	5	6	7
1	Trait competitiveness	4.75	1.35	(.89)						
2	Competitive climate	3.35	0.84	.48	(.75)					
3	Dark Triad composite	2.25	0.75	.34	.19	(.88)				
4	Machiavellianism	2.48	0.93	.26	.21	.87	(.80)			
5	Psychopathy	2.77	0.75	.12	.01	.81	.61	(.78)		
6	Narcissism	2.19	0.91	.45	.25	.80	.55	.42	(.80)	
7	Positive attitude	2.07	0.90	.02	.04	.26	.25	.18	.21	(.87)

Correlations of with a value of .11 and higher are significant at *p* < .05. Cronbach Alpha is reported in parentheses

The DT was measured using the Dirty Dozen (4 items per dimension [82]). Sample items included: for Machiavellianism, “I tend to manipulate others to get my way”; for psychopathy, “I tend to lack remorse”; for narcissism, “I tend to want others to admire me”. We also computed an aggregated score for the whole instrument (DT composite index).

Trait competitiveness was measured using four items from the Work and Family Orientation Scale [83]. Our four-item measure of competitive climate was adapted from [83] by generalizing the wording of some items of the Perceived intra-organizational competition scale beyond the sales context. sample items included “I enjoy working in situations involving competition with others” for Trait competitiveness, and “My co-workers frequently compare their results with mine” for Competitive climate.

Our criterion variable, general attitude towards PCE, was measured using a 9-item scale that we developed from reviewing the literature on expert opinion about PCE. Sample items included “In our society PCE is a step in the right direction of development”, “PCE should be incorporated in workplace situations”. The measurement reflects a positive general attitude towards PCE.

We included socio-demographic variables in the survey: gender, age, employment status (employed full time, employed part time, unemployed looking for work, unemployed not looking for work, retired, student, disabled) and educational attainment (Less than high school; High school graduate, 2 year degree, 4 year degree, Professional degree, Doctorate).

### Data analysis

The data were analysed in R. We normalized all predictors in order to avoid multicollinearity in testing moderations. All variance inflation factor values were below the recommended upper threshold of 4 (no *VIF* exceeded 1.98), thereby showing no multicollinearity issue.

The tests of Shapiro–Wilk (*W* = 0.983, *p* < 0.001) and Kolmogorov–Smirnov (*K* = 0.063, *p* = 0.14) – performed using the package *olsrr*—led to divergent results regarding the normality of the residuals for the models testing our hypotheses. QQ plots further showed only minute differences from expected values under conditions of normality of residuals. We therefore present complete results from ordinary least squares regression using the function *lm()* and ANCOVAs with the base function *aov()* in the main text, as well as, in “Appendix B”, results from robust analyses (Tables 4 and 5) using the package *robust* with functions *lmRob()* for robust regressions and *Ancova.lmRob()* for robust ANCOVAs. These results are also summarized in the main text.

## Results

Zero-order correlations between the study variables and descriptive statistics are presented in Table 1. Positive attitude toward PCE was positively associated with the DT composite index and all DT subscales but was not associated with trait competitiveness nor competitive climate. Trait competitiveness was positively associated with competitive climate, the DT composite index and all DT subscales. Competitive climate was positively associated with the DT composite index, and two of the DT subscales: Machiavellianism and narcissism. The DT composite was positively associated with all DT subscales.

### Dark Triad

We hypothesized that the DT composite and its dimensions would be positively associated with positive attitudes towards PCE. Results from the multiple regression analyses (OLS), which were performed separately for the DT composite index (Model 1; *F*(3, 222) = 8.228, *p* < 0.001; Table 2) and its dimensions (Model 2; *F*(5,320) = 5.263, *p* = < 0.001, were consistent with correlations in the case of the DT composite index (H1, *B* = 0.211; *p* < 0.001). The ANCOVA also confirmed this hypothesis, *F*(1,322) = 24.113, *p* < 0.000.

**Table 2:** Multiple regression analyses and ANCOVAs without moderators with positive attitude as the criterion variable (*N* = 326)

	Model 1a			Model 2a		
	*B*	*t*	*F*	*B*	*t*	*F*
Intercept	2.774	68.579***		2.774	68.543***	
Trait competitiveness	− 0.063	− 1.330	0.175	− 0.070	− 1.408	0.174
Competitive climate	0.020	0.441	0.396	0.009	0.202	0.395
Dark Triad composite	0.211	4.91***	24.113***			
Machiavellianism				0.136	2.390***	21.669***
Psychopathy				0.020	0.387	0.463
Narcissism				0.101	1.901^#^	3.612^#^
	*R2*			*R2*		
	.071			.067		

The terms in the ANCOVAs (df = 3322 for each predictor in Model 1a and df = 5320 in Model 2a) were included sequentially

^#^*p* < .10; *p < .05; **p < .01; ***p < .001

In multivariate analyses, only Machiavellianism (H2, *B* = 0.136; *p* = 0.017; *F*(1, 320) = 21.668, *p* < 0.001) was significantly and positively related to positive attitudes towards PCE. Narcissism was only marginally and positively related to positive attitudes towards PCE (H4; *B* = 0.101, *p* = 0.058; *F*(1,320) = 3.612, *p* = 0.058) and psychopathy had no effect on positive attitudes towards PCE (H3). Results from robust analyses (see Table 4 in “Appendix B”) confirmed such effect of the DT composite measure on positive attitudes towards PCE (robust*B* = 0.215, *p* < 0.001; robust*F*(1,322) = 21.832, *p* < 0.001). The robust ANCOVA not only confirmed the effect of Machiavellianism (H2), robust*F*(1, 322) = 17.549, *p* < 0.001, but also highlighted a significant effect of narcissism (H4), robust*F*(1, 322) = 3.939, *p* = 0.043. Yet the robust regression coefficients were non-significant. No effect of psychopathy was found in robust analyses neither (H3).

### Competitiveness

We hypothesized that Trait competitiveness and Competitive climate would be positively related to Positive attitudes towards PCE (H5 and H6). Neither correlations nor results from any of our other analyses supported these hypotheses at *p* < 0.05, but a marginal effect was found in robust ANCOVAs (*F*(1, 320) = 3.031, *p* = 0.07) for competitive climate.

### Moderation analyses

**Fig. 1:**
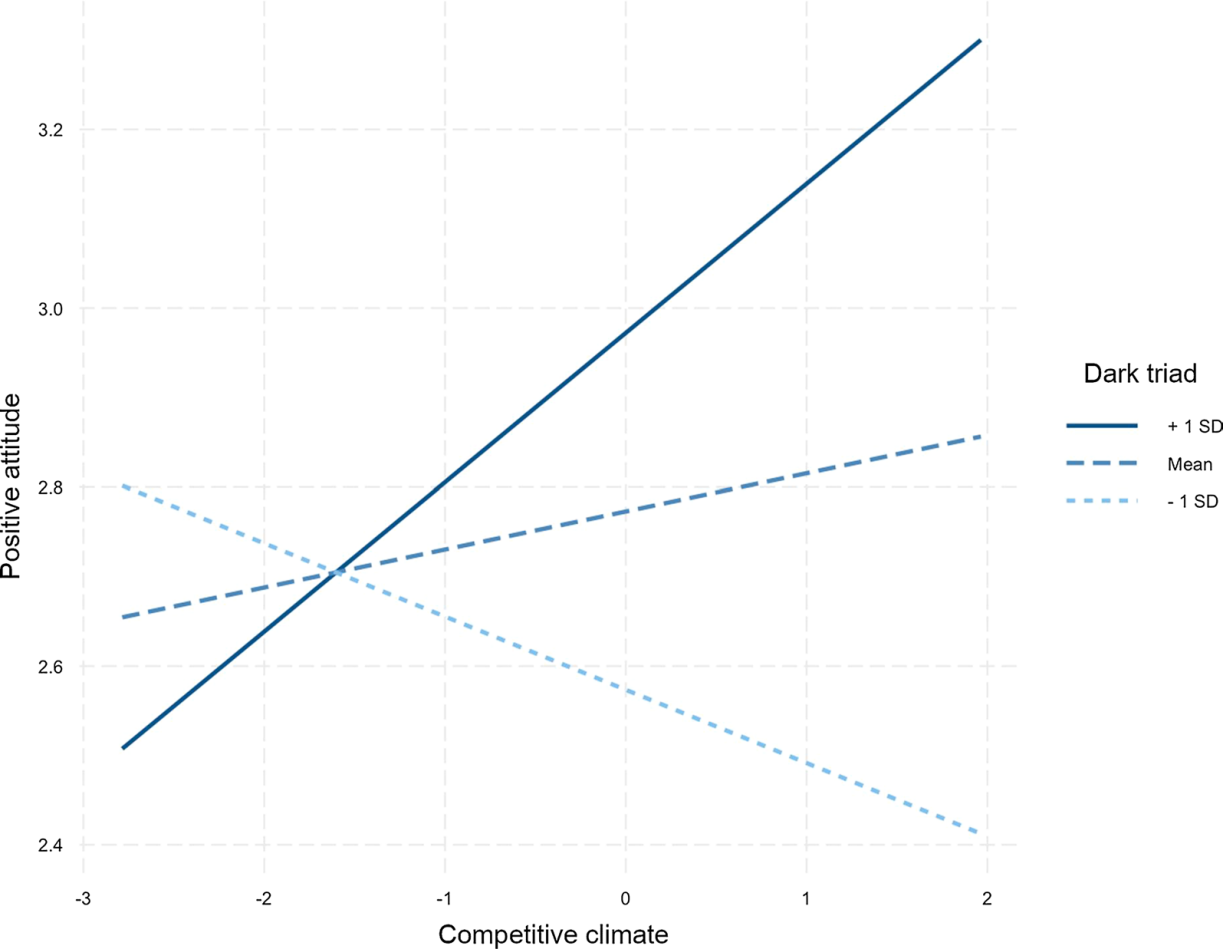
Interaction plot of the moderated effect of competitive climate on positive attitudes towards CE by the DT composite measure

We hypothesized that the DT composite measure would be a positive moderator of the relationships between (a) trait competitiveness and positive attitudes towards PCE and (b) competitive climate and positive attitudes towards PCE (H7). The results for OLS are presented in Table 3. The model testing the moderating effect of the DT composite measure (Model 1b) predicted significant additional variance, *F*Change(2, 320) = 3.315, *p* = 0.038. The effect of competitive climate on positive attitudes was indeed moderated by the Dark Triad composite measure in OLS analyses (*B* = 0.124, *p* = 0.01). Figure 1 displays this moderating effect, which supports our hypothesis of a stronger effect of competitive climate on positive attitudes towards PCE in individuals with a high score on the DT composite measure. The model including the DT dimensions as moderators (H8 to H10) failed to explain additional variance, as did our robust regression models including moderations (Table 5 in “Appendix B”).

**Table 3:** Multiple regression analyses and analyses of covariance (ANCOVAs) with positive attitude as the criterion variable (*N* = 326)—moderators involved

	Model 1b			Model 2b		
	*B*	*t*	*F*	*B*	*t*	*F*
Intercept	2.773	65.679***		2.784	62.638***	
Trait competitiveness (TC)	− 0.073	− 1.448	0.177	− 0.078	− 1.453	0.176
Competitive climate (CC)	0.043	0.913	0.401	0.033	0.681	0.398
Dark Triad composite (DT)	0.199	4.556***	24.460***			
TC * DT	− 0.067	− 1.380	0.048			
CC * DT	0.124	2.566*	6.582*			
Machiavellianism (M)				0.089	1.185	21.819***
Psychopathy (P)				0.087	1.255	0.466
Narcissism (N)				0.106	1.476	3.637^#^
TC * M				− 0.090	− 1.204	0.116
TC * P				− 0.034	− 0.479	0.967
TC * N				− 0.008	− 0.115	0.008
CC * M				0.001	0.009	2.182
CC * P				0.104	1.646*	4.521*
CC * N				0.014	0.210	0.417
	*R2*	*Fchange*		*R2*	*Fchange*	
	0.089	3.315*		0.99	1.684	

The *FChange* value is computed in comparison with the models without moderators (Table 2). The terms in the ANCOVAs were included sequentially

^#^*p* < .10; *p < .05; **p < .01; ***p < .001

## Discussion

The use of cognitive enhancement is frown upon and considered unethical by most people (e.g., [28]). While acknowledging the importance of public attitudes towards PCE (e.g., [26, 37]), the literature has mostly focused on ethical considerations, and untested theorization, such as proposing the contextual role of competition in predicting interest in PCE (e.g., [84]). Therefore, research has overlooked the role of individual differences, notably personality, in the formation of these attitudes and determinants of dissident opinions have only been scarcely investigated. The present study contributed to such enquiry.

We investigated for the first time whether general attitudes towards PCE were related to DT, as well as to individual and contextual competitiveness. Results of analyses of (robust) regression and (robust) ANCOVAs showed that the DT composite index (H1) was significantly related to positive general attitudes towards PCE. OLS regression results and (robust) ANCOVAs confirmed the association of Machiavellianism (H2) with positive attitudes towards PCE. A significant association of narcissism with such attitudes was found in the robust ANCOVA, yet this association was marginally significant in the OLS regression, the ANCOVA and the robust regression (H4). A positive association between psychopathy and positive attitudes towards PCE was found when assessed in a correlation test (H3), but not in (robust) regression analysis nor (robust) ANCOVAs.

We also hypothesized positive associations between competitiveness, both individual (trait competitiveness; H5) and contextual (competitive climate; H6), and positive attitudes towards PCE. These hypotheses were not confirmed.

Finally, this study is also the first to examine the moderating role of DT and its dimensions on the associations between positive attitudes towards PCE and individual and contextual competitiveness. Results from OLS regression and (robust) ANCOVAs have highlighted that the effect of contextual competitiveness on attitudes towards cognitive enhancement might only be present in individuals with high endorsement of DT (H7).

### Implications for the Dark Triad literature

The literature on the DT has shown its dimensions were related to undesirable behaviours and unconventional ethical positions [9, 57, 60, 61]. Our results (H1 to H4) suggest that the dimensions of DT have a differentiated influence on the attitudes towards cognitive enhancement. It is worth noting that similar patterns of results have been found with regards to other undesirable outcomes, such as high stakes deception [57], fraudulent behavior [85], or unethical decision-making [60], for which the large contribution of Machiavellianism to explained variance has been robustly highlighted.

Contextual factors are known to influence the formation of attitudes and the production of behaviour [4], yet individual differences might affect such influences [86]. Indeed, research investigating unethical decision-making has highlighted the relevance of an interactionist perspective taking into account the confluence of individual variables and contextual or situational factors in detecting fraud [87]. Yet, the moderating role of the DT on the effects of a contextual factor has rarely been investigated. An investigation of such moderating effects found that the effect of transformational leadership on unethical pro-organizational follower behavior was moderated by Machiavellianism [88]. In another study, the DT was found to moderate the effect of workplace spirituality on incivility from superiors and colleagues [89]. Following this line of research, our study shows (H7) that individuals working in competitive climates might be more receptive to PCE if they are highly manipulative (Machiavellianism), have a pattern of entitlement and grandiosity (narcissism), and look for personal gain at the expense of others and without remorse (psychopathy).

### Implications for the cognitive enhancement (and related) literature

Nicholls and colleagues [63] found all DT dimensions were linked to positive attitudes towards doping in sports. The distinctiveness of the views towards cognitive enhancement and doping in sports is an important research avenue [29]. Use by employees has been reported to be more acceptable than use by students or athletes [31]. In this study, DT dimensions explained 29% of the variance in positive attitudes towards doping in sports [31]. But in our study, the DT explained only 7% of the variance in positive attitudes towards PCE. One interpretation could be that these results have highlighted additional differences between the two concepts in their relationship with the DT. Another explanation of these findings could be related to the lower familiarity of CE compared with doping. Even though cognitive enhancement has been vastly discussed in the media in the recent years, its side effects have only infrequently on been mentioned [4–6]. Individuals in 2012 were indeed quite unfamiliar with CE [29]. This could attenuate the relationships between DT and positive attitudes towards PCE, as limited knowledge of the side-effects could make CE less abhorrent to people in general and therefore allow less variance to be predicted by the DT. But, contrary to this explanation, familiarity with CE is positively related with favourable views of CE [29, 40].

### Practical implications

Targeting at risk groups could render public health campaigns more effective [3]. Research has shown that career preferences are, in part, a function of DT [90]. For instance, Machiavellians prefer jobs in the financial and law industries and in the domain of management [91]. It might be desirable to first conduct studies focusing on attitudes towards PCE in these professions in order to help design effective prevention campaigns.

Authors have advocated for including the public in debates relating to cognitive enhancement [8, 20]. Our findings show that the composition of discussion groups in terms of individuals’ Machiavellianism could potentially affect the outcomes of such debates.

### Limitations

This study relied on cross-sectional data. The direction of the relationships among the variables of interest in such datasets is usually difficult to establish. We believe that this is less the case here because of the constructs of interest: It is unlikely that the attitude towards PCE would influence the development of DT. The relevance of the hypothesized direction of causality (DT influencing attitudes) has been explained above from a theoretical point of view, yet longitudinal studies are necessary to empirically ascertain that attitudes towards PCE do not foster the development of DT.

## Conclusion

The use of pharmacological cognitive enhancers has increased in recent years, despite the fact that such substances can have health-threatening side-effects. Policy-making as well as the research literature on cognitive enhancement can benefit from a better understanding of what drives positive attitudes towards PCE. Our study has contributed to this enquiry in 2 main ways. On the one hand, we have shown that attitudes towards cognitive enhancers are in part driven by dark personality traits. On the other hand, we found that competitive climate may increase positive attitudes towards PCE, but only in individuals scoring high on dark personality traits.

## Data Availability

The data used in this manuscript are available at: https://osf.io/3vfgu/?view_only=04b5fba3bf20498aa2ff243ddbe7f1e3

## Appendix A

### Socio-demographic variables

Age

Numeric

Gender

*Response choices*:

Male, Female

Employment status

*Response choices*:

Employed full time, Employed part time, Unemployed looking for work, Unemployed not looking for work, Retired, Student, Disabled

Educational attainment

*Response choices*:

Less than high school, High school graduate, 2 year degree, 4 year degree, Professional degree, Doctorate

### Measured constructs

Trait competitiveness [83]

*Response choices*:

Strongly disagree, Disagree, Somewhat disagree, Neither agree nor disagree, Somewhat agree, Agree, Strongly agree

*Items*:

I feel that winning is important in both work and games.

I enjoy working in situations involving competition with others.

It is important to me to perform better than others on a task.

I try harder when I am in competition with others.

Competitive climate [83; adapted]

*Response choices*:

Strongly disagree, Somewhat disagree, Neither agree nor disagree, Somewhat agree, Strongly agree

*Items*:

The amount of recognition your get in a company depends on how your performance ranks compared to other colleagues.

My manager frequently compares my results with those of other colleagues.

My coworkers frequently compare their results with mine.

Everybody is concerned with finishing at the top of the performance ratings.

Dirty Dozen Dark Triad [82]

*Response choices*:

Strongly disagree, Somewhat disagree, Neither agree nor disagree, Somewhat agree, Strongly agree

*Machiavellianism items*:

I tend to manipulate others to get my way.

I have used deceit or lied to get my way.

I have use flattery to get my way.

I tend to exploit others towards my own end.

*Psychopathy items*:

I tend to lack remorse.

I tend to not be too concerned with morality or the morality of my actions.

I tend to be callous or insensitive.

I tend to be cynical.

*Narcissism items*:

I tend to want others to admire me.

I tend to want others to pay attention to me.

I tend to seek prestige or status.

I tend to expect special favors from others.

Attitude towards PCE (self-developed scale)

*Response choices*:

Strongly disagree, Somewhat disagree, Neither agree nor disagree, Somewhat agree, Strongly agree.

*Items*:

PCE reduces the value of performance.

PCE is an adequate option if a task is too difficult.

PCE induces more cooperation in society.

PCE is without danger.

PCE reduces the authenticity of their users’ contribution.

It is morally wrong to use PCE, even if something important is at stake.

In our society, PCE is a step in the right direction of development.

PCE should be incorporated in workplace situations.

The use of PCE does not threaten health.

## Appendix B

See Tables 4 and 5.

**Table 4:** Robust multiple regression analyses and robust analyses of covariance (ANCOVAs) with positive attitude as the criterion variable (*N* = 326)―no moderators involved

	Model 1a			Model 2a		
	Robust *B*	Robust *t*	Robust *F*	Robust *B*	Robust *t*	Robust *F*
Intercept	2.761	56.51***		2.766	56.784***	
Trait competitiveness	− 0.028	− 0.477	2.459	− 0.033	− 0.541	2.459
Competitive climate	0.052	0.937	3.031^#^	0.051	0.904	3.031^#^
Dark Triad composite	0.215	4.058***	21.833***			
Machiavellianism				0.095	1.384	17.549***
Psychopathy				0.048	0.762	1.276
Narcissism				0.116	1.765^#^	3.939*
	*R2*			*R2*		
	.071			.067		

The terms in the robust ANCOVAs were included sequentially

^#^*p* < .10; **p* < .05; ***p* < .01; ****p* < .001

**Table 5:** Robust multiple regression analyses and robust ANCOVAs with moderators with positive attitude as the criterion variable (*N* = 326)

	Model 1b			Model 2b		
	Robust B	Robust t	Robust F	Robust B	Robust t	Robust F
Intercept	2.780	51.884***		2.790	49.155***	
Trait competitiveness (TC)	− 0.057	− 0.909	2.459	− 0.054	− 0.810	2.459
Competitive climate (CC)	0.069	1.177	3.031^#^	0.063	1.047	3.031^#^
Dark Triad composite (DT)	0.224	3.941***	21.833***			
TC * DT	− 0.113	− 1.811^#^	2.293			
CC * DT	0.101	1.622	4.209*			
Machiavellianism (M)				0.089	1.185	17.549***
Psychopathy (P)				0.087	1.255	1.276
Narcissism (N)				0.106	1.476	3.939*
TC * M				− 0.090	− 1.204	4.342*
TC * P				− 0.034	− 0.479	0.178
TC * N				− 0.008	− 0.115	0.078
CC * M				0.001	0.009	0.908
CC * P				0.104	1.646	5.073*
CC * N				0.014	0.210	0.142
	*R2*	*Fchange*		*R2*	*Fchange*	
	0.089	3.188		0.1	1.684	

The *FChange* value is computed in comparison with the models without moderators (Table 4). The terms in the robust ANCOVAs were included sequentially

^#^*p* < .10; **p* < .05; ***p* < .01; ****p* < .001

## Abbreviations

CE: Cognitive enhancement
DT: Dark Triad of personality
NPC: Non-pharmacological cognitive enhancement
PCE: Pharmacological cognitive enhancement
